# Impact of DNA Damage Response—Targeted Therapies on the Immune Response to Tumours

**DOI:** 10.3390/cancers13236008

**Published:** 2021-11-29

**Authors:** Nura Lutfi, Miguel Alejandro Galindo-Campos, José Yélamos

**Affiliations:** 1 Cancer Research Program, Hospital del Mar Medical Research Institute (IMIM), 08003 Barcelona, Spain; nlutfi@imim.es (N.L.); mgalindo@imim.es (M.A.G.-C.); 2 Immunology Unit, Department of Pathology, Hospital del Mar, 08003 Barcelona, Spain

**Keywords:** DNA damage response-targeted agents, immune response

## Abstract

**Simple Summary:**

Targeting tumour-specific defects in the DNA damage response (DDR) presents an opportunity for new therapeutic approaches to selectively kill cancer cells. Although the therapeutic rationale of DDR-targeted agents initially focused on their actions against tumour cells, these agents might also alter the crosstalk between tumour cells and the immune system. Here, we discuss recent data showing that DDR-targeted agents affect the antitumour immune response both through direct actions on the immune system components and through indirect effects on expression of different molecules and pathways in tumour cells that underpin the tumour cell–immune system.

**Abstract:**

The DNA damage response (DDR) maintains the stability of a genome faced with genotoxic insults (exogenous or endogenous), and aberrations of the DDR are a hallmark of cancer cells. These cancer-specific DDR defects present new therapeutic opportunities, and different compounds that inhibit key components of DDR have been approved for clinical use or are in various stages of clinical trials. Although the therapeutic rationale of these DDR-targeted agents initially focused on their action against tumour cells themselves, these agents might also impact the crosstalk between tumour cells and the immune system, which can facilitate or impede tumour progression. In this review, we summarise recent data on how DDR-targeted agents can affect the interactions between tumour cells and the components of the immune system, both by acting directly on the immune cells themselves and by altering the expression of different molecules and pathways in tumour cells that are critical for their relationship with the immune system. Obtaining an in-depth understanding of the mechanisms behind how DDR-targeted therapies affect the immune system, and their crosstalk with tumour cells, may provide invaluable clues for the rational development of new therapeutic strategies in cancer.

## 1. Introduction

The genome is exposed to a multitude of genotoxic insults, both exogenous (e.g., mutagenic chemicals, ionising radiation) and endogenous (e.g., reactive oxygen species), that generate DNA damage. Moreover, endogenous DNA damage can lead to generation of recombination intermediates and arrested forks. To preserve genomic integrity, all eukaryotic cells have evolved mechanisms that sense DNA lesions, signal their presence and then promote their repair. Concomitant to DNA break repair, a rapid signalling cascade must be coordinated at the lesion site, which leads to activation of cytostatic and cytotoxic responses that limit the expansion of the damaged cells. Collectively, these mechanisms are referred to as the DNA damage response (DDR) [1]. Aberrations in the DDR result in genomic instability, which is a hallmark of cancer cells but at the same time opens up cancer treatment opportunities by targeting tumour-specific DDR defects to selectively kill cancer cells [2].

**Figure 1:**
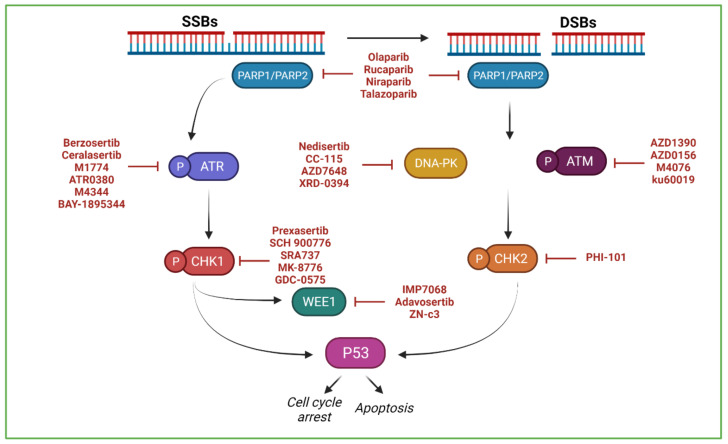
DDR-targeted agents that have been clinically approved or are in clinical trials. PARP-1 and PARP-2 become enzymatically active upon recognition of DNA breaks, cleave β-NAD^+^ and transfer ADP-ribose moieties (PARylation) onto specific amino acid residues of acceptor proteins. The kinases ATR and ATM phosphorylate hundreds of proteins, including CHK1 and CHK2, respectively, which are key mediators of the checkpoint function. Whereas ATM is activated by DNA double-strand breaks (DSBs), ATR is activated by DNA single-strand breaks (SSBs). The kinase DNA-PK is critical for DSB repair via the non-homologous end-joining pathway. The kinase WEE1 controls mitotic entry.

Poly(ADP-ribose) polymerases inhibitors (PARPi) that target both the PARP-1 and PARP-2 enzymes (e.g., olaparib, niraparib, rucaparib and talazoparib) have been the first DDR-targeting compounds approved for treatment of some tumours bearing homologous recombination (HR) DNA repair deficiencies [3], acting on the principle of synthetic lethality [4,5]. Other critical components of the DDR, including ataxia telangiectasia and Rad3-related (ATR), ataxia telangiectasia mutated (ATM), DNA-dependent protein kinase (DNA-PK), Checkpoint kinase 1 (CHK1), Checkpoint kinase 2 (CHK2) and Wee1 kinase (WEE1), are also promising as targets for fighting cancer [6,7]. Compounds that aim to inhibit these proteins are now at different phases of clinical development as either monotherapies or in combination with other therapies, mainly immunotherapies; it is expected that some of them will be approved for clinical use in the short term [6,8] (clinicaltrials.gov; accessed on 1 October 2021) (Figure 1).

These DDR-targeting agents have mainly been considered for their intrinsic actions towards tumour cells per se. However, these compounds will also affect the immune response to tumours, both by acting directly on the components of the immune system and by modifying components and signalling pathways in tumour cells that shape their interactions with the immune system. In this review, we summarise recent data on how DDR-targeted agents affect the antitumour immune response, focusing mainly on clinically approved compounds. Understanding how DDR-targeted therapies affect (i) the immune system and (ii) the crosstalk between the immune system and tumour cells may provide invaluable clues for designing new (and refining existing) therapeutic strategies in cancer.

## 2. Effects of DDR-Targeted Therapies on the Cell Components of the Immune System

The initial design of DDR-targeting agents to selectively kill tumour cells focused on their effects on tumour cells per se. However, systemic administration of these agents is likely to also impact the immune system, which may affect their therapeutic actions by facilitating or impeding tumour progression. This systemic effect on the immune system affects both the immune cells present in the tumour microenvironment (TME) and the peripheral immune cells that are required to drive effective antitumour immune responses [9,10,11].

Interestingly, in preclinical mouse models, treatment with the PARPi olaparib over a short course results in depletion of reticulocytes, B-cell progenitors and immature thymocytes, while longer treatment results in myelosuppression [12]. Moreover, patients treated with PARPi show a variety of adverse effects, including lymphopenia and other haematological toxicities [13,14].

Recently, we observed that olaparib treatment significant decreases tumour growth when breast cancer AT-3 cells, which are sensitive to olaparib, are implanted into an immunodeficient syngeneic mouse model but not if they are implanted into immunocompetent mice, suggesting that the antitumour effect of olaparib on the AT-3 cancer cells competes with the potentially negative pro-cancer effect of olaparib on the immune system [15]. However, other evidence suggests that DDR-targeted therapies may increase the antitumour immune response through several different mechanisms, such as by increasing antigenicity, increasing the gene instability of tumour cells, activating cytosolic immunity or modulating different components that may affect interactions between tumour and immune cells [16]. Together, these different aspects of the DDR effect on the immune system highlight the complex relationships between cancer and immune cells.

**Figure 2:**
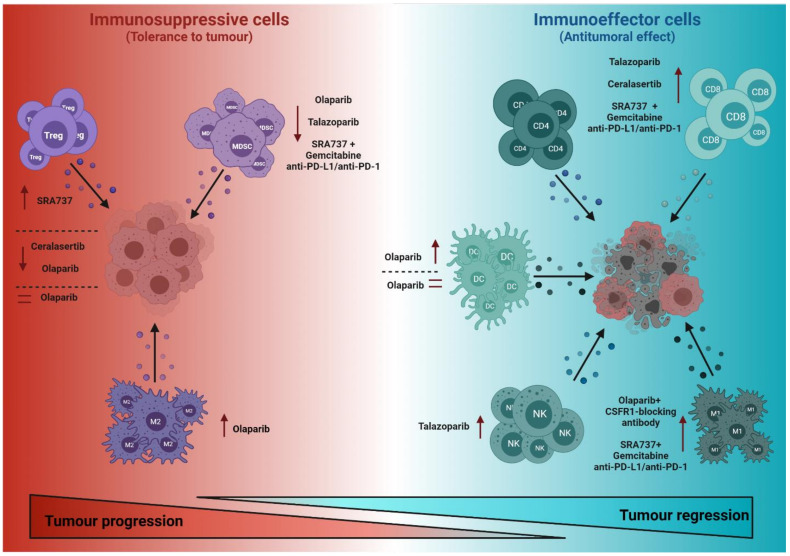
Effects of DDR-targeted agents on the cell components of the immune system. DDR-targeted agents can affect different cells of the immune system, either with immunosuppressive functions (left) or effector functions (right), and thus contribute to promoting or inhibiting tumour progression. CSF1R, colony-stimulating factor 1 receptor; M1, M1 macrophages; M2, M2 macrophages; MDSC, myeloid-derived suppressor cells; NK, natural killer cell; PD-1, programmed death protein-1; PD-L1, programmed death ligand-1; Treg, regulatory T cells.

Immune cells in the TME include both those associated with immunological tolerance to tumours (immunosuppressive function) and immune effector cells against tumours (antitumour function). Understanding the impact of DDR-targeted agents in the delicate balance between these two different functional immune cell types will be critical for learning how to regulate tumour fate (Figure 2).

### 2.1. Effects of DDR-Targeted Therapies in Cells with Immunosuppressive Functions

The effects of DDR-targeted agents on the recruitment and function of cells with immunosuppressive functions—and therefore tumour progression-promoting functions—are beginning to be understood. The main cells with immunosuppressive functions are myeloid-derived suppressor cells (MDSCs), M2-macrophages and regulatory T cells (Tregs).

MDSCs represent a heterogeneous group of immature myeloid cells associated with immunosuppressive functions in the TME that contribute to tumour progression [17]. Accordingly, interfering with their recruitment to the TME and/or blocking their functional activity may improve the immune response to tumours. For instance, olaparib treatment suppresses MDSC migration to the TME [18]. The mechanisms underlying this effect appear to be based on olaparib downregulating the expression of stromal cell-derived factor 1-alpha (SDF1α), which is released to the TME by cancer-associated fibroblasts (CAF) and reduces CXCR4-mediated MDSC migration [18]. Interestingly, this olaparib-mediated decrease of infiltrating MDSCs in the TME improved the therapeutic efficacy of chimeric antigen receptor (CAR)-T cells in a mouse model of breast cancer [18]. Likewise, using the PARPi talazoparib encapsulated in a bilayer of a nano-liposome also decreased the levels of MDSCs, in both tumour and spleen in a mouse model of breast cancer [19]. Similarly, olaparib treatment reduced the granulocyte component of the MDSC population in a mouse model of breast cancer susceptibility genes 1 (BRCA1)-deficient ovarian cancer [20]. Strikingly, in a colon cancer model, moderate doses of olaparib only had a minor impact on the migration of MDSCs into the tumour, but inhibited the immunosuppressive activity of intratumour MDSCs and enhanced the response to anti-PD-1 therapy [21]. In addition to PARPi, treatment with a combination of the CHK1i SRA737 and the antimetabolite agent gemcitabine plus anti-PD-L1/anti-PD-1 also decreased MDSCs in a small cell lung cancer (SCLC) model [22].

Macrophages differentiate from circulating monocytes after extravasation to tissues and play different roles, including tissue homeostasis, host defence, antigen presentation to T cells and modulation of inflammatory responses [23]. A characteristic feature of macrophages is their extensive plasticity, which allows them to adapt their phenotype in response to environmental cues [24]. Tumour-associated macrophages (TAMs) are classified into two main groups, according to their antitumour (M1) or pro-tumour (M2) properties [23]. Of note, olaparib treatment in combination with radiotherapy results in macrophage activation towards a proinflammatory M1 phenotype in a colorectal tumour model [25]. However, in a mouse model of BRCA1-deficient, triple-negative breast cancer (TNBC), olaparib treatment had a dual effect on macrophages, with an antitumour effect due to inducing macrophage expression of co-stimulatory molecules (CD80) and activation markers (CD40), and a pro-tumour effect due to increasing the expression of immunosuppressive markers (e.g., programmed death ligand-1, PD-L1) and colony-stimulating factor 1 receptor (CSF1R) [26]. Combining olaparib treatment with macrophage-targeting therapy by CSF1R-blocking antibodies significantly enhances the antitumour immune response and increases survival in mice with BRCA-deficient TNBC tumours [26]. In addition, treatment with a combination of the CHK1i SRA737, gemcitabine and anti-PD-L1/anti-PD-1 increased M1 macrophages and decreased M2 macrophages in a SCLC model [22].

Tregs comprise a subpopulation of T lymphocytes with immunosuppressive functions and therefore impaired immune response to tumours [27]. Previous studies have determined that mice deficient for PARP-1 have increased levels of Tregs [28,29], which may have contributed at least in part to the acceleration of tumourigenesis in the absence of PARP-1 in a c-Myc-driven B cell lymphoma mouse model [30]. However, despite the critical role of Tregs in modulating the immune response, studies about the effects of DDR-targeted agents on Tregs are very limited and show varying results. For instance, olaparib treatment reduced the increase in Treg levels observed in a mouse model of sepsis [31]. Nevertheless, in a BRCA1-deficient mouse model of ovarian cancer, the percentage of the intratumour Treg cell population was not modified after olaparib treatment [20]. In contrast, tumour-infiltrating Tregs were increased in a mouse model of melanoma treated with the CHK1i SRA737 plus a subclinical dose of hydroxyurea [32]. A combination therapy of the ATRi ceralasertib and radiation decreased the number of tumour-infiltrating Tregs in mouse models of KRAS-mutant cancer [33], as well as in a mouse model of hepatocellular carcinoma [34]. Altogether, these findings show the need for further studies that evaluate Tregs in patients receiving DDR-targeted agents.

### 2.2. Effects of DDR-Targeted Therapies on Immune Cells with Antitumoural Effector Functions

CD8 cytotoxic T cells and CD4 Th1 cells are the most important contributors to the adaptive immune cellular host defence against tumours [35]. T cell recognition of tumour antigens requires that these are processed by antigen-presenting cells (APCs)—mainly dendritic cells (DCs)—that present them in the context of self-major histocompatibility complex (MHC) molecules to naïve T cells in order to prime them [36]. This antigenic presentation, together with co-stimulatory signals, results in CD8 and CD4 naïve T cells differentiating into effector cells with antitumour activity. The antitumour functions of CD8 T cells are primarily mediated by the secretion of perforin and granzyme [37], whereas those of CD4 T cells are primarily mediated by the secretion of various cytokines such as IL-2 and tumour necrosis factor (TNF) that enhance the CD8 T cell response and activate other effector cells, such as natural killer (NK) cells [38].

Of note, we have previously demonstrated that dual deficiency for PARP-1 and PARP-2 in T cells results in a significant decrease of both CD4 and CD8 peripheral T cells [29] and consequently impairs the T cell response to tumours [15]. Although it seems clear that PARPi will not achieve the degree of inhibition that can be achieved by genetic deletion of PARP-1 and PARP-2, these inhibitors can affect the T cell compartment, which could compromise the T cell immune response to tumours. In this sense, it would be of interest to evaluate the T cell compartment (effector CD8 and CD4 vs. naïve) in PARPi-treated patients.

Most pre-clinically reported effects of DDR-targeted therapies on antitumour effector T cells have been attributed to the effects of these inhibitors on the tumour cell itself. For instance, therapies can activate the cGAS/STING pathway, thereby indirectly modulating T cell responses by modifying the relationship between the tumour and T cells (see also below). Nevertheless, some data have been obtained on the direct effects of DDR-targeted agents on T cells in which the cGAS/STING pathway does not play a role (or the role has not yet been determined). For example, the PARPi talazoparib significantly increased the number of peritoneal CD8 T cells as well as their production of interferon-γ (IFN-γ) and TNF-α in a mouse model of ovarian cancer [39]. Moreover, the activity of cytotoxic CD8 T cells and NKT cells increased in a mouse model of melanoma treated with the CHK1i SRA737 plus subclinical dose of hydroxyurea, in a cGAS/STING-independent manner [32]. The CHK1i (SRA737) in combination with gemcitabine and anti-PD-L1/anti-PD-1 increased antitumor CD8 cytotoxic T cells in a SCLC model [22]. In mouse models of KRAS-mutant cancer, the ATRi ceralasertib attenuated radiation-induced CD8 T cell exhaustion and potentiated CD8 T cell activity [33].

DCs play a critical role in the immune response against tumour cells through their role in antigen presentation to T cells, yet the effects of DDR-targeted therapies on their function is largely unknown. Ding et al. (2018) found that olaparib treatment increased levels of co-stimulatory effects of CD80 and CD86 molecules as well as of MHC-class II molecules on DCs, suggesting increased costimulatory and antigen-presentation activity to T cells [20]. In contrast, another study showed that olaparib does not affect DC differentiation or function [40]. These data indicate the need for further studies to clarify the effects of DDR-targeted agents on DC function and the impact this has on the antitumour immune response.

NK cells are components of the innate immune system and have an important role in the antitumour response, based on several of their functions: (i) their cytolytic activity via the release of perforin and granzyme; (ii) inducing apoptosis in target cells via the production of TNF; (iii) inducing apoptosis via cell–cell contact and activation of the tumour necrosis factor-related apoptosis-inducing ligand (TRAIL) and Fas ligand (FASL) pathways [41,42]; and (iv) killing tumour cells coated with antibodies via antibody-dependent cell-mediated cytotoxicity (ADCC) [43]. Many of the effects induced by DDR-targeted agents on the antitumour activities of NK cells described so far seem to be due to modifications of tumour cells themselves, which affect their interactions with NK cells (see below). Indeed, whether DDR-targeted compounds have any direct effects on NK cells themselves is largely unknown. Of note, treatment with the PARPi talazoparib significantly increased the number of peritoneal NK cells as well as their production of IFN-γ and TNF-α in a mouse model of ovarian cancer [39].

## 3. DDR-Targeted Agents Modulate Pathways in Tumour Cells That Impact Their Relationships with Immune Cells

**Figure 3:**
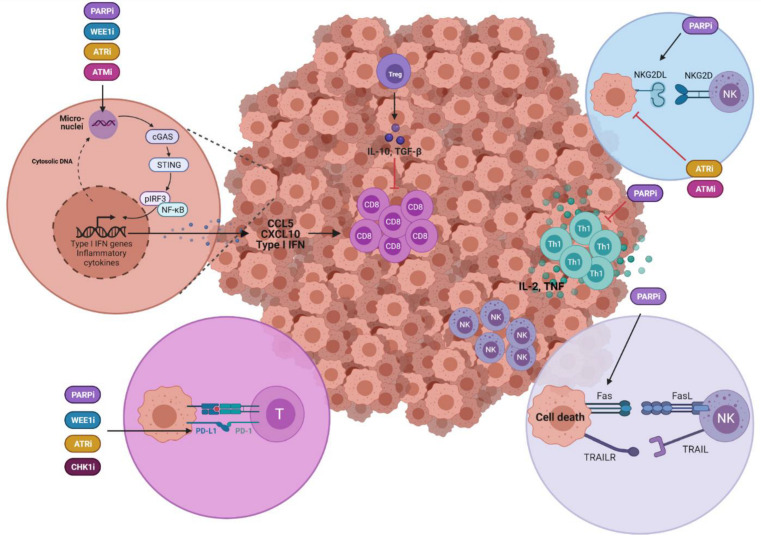
Schematic representation of the tumour microenvironment depicting the effects of DDR-targeted agents on the crosstalk between tumour cells and infiltrating immune cells. cGAS, GMP-AMP (cGAMP) synthase; IFN, interferon; NK, natural killer cell; NKG2DL, NKG2D ligand; PD-1, programmed death protein-1; PD-L1, programmed death ligand-1; pIRF3, phosphor IFN regulatory factor 3; STING, stimulator of IFN genes; Treg, regulatory T cells; TRAILR, TRAIL receptor.

DDR-targeted agents modulate different molecules and pathways in tumour cells that are critical in their relationships with the immune system; in this way, they may either limit or favour tumourigenesis (Figure 3).

### 3.1. DDR-Targeted Agents Modulate Immune Responses via Activation of the cGAS/STING Pathway in Tumour Cells

Recent data have shown that PARPi promotes accumulation of cytosolic DNA [44,45], via PARP-1 trapping-induced DDR [46]. This often results in micronuclei that can arise from unresolved genomic instability, from either lagging chromosomes and/or chromatin bridges [47,48]. ATRi treatment further increases the numbers of PARPi-induced micronuclei in BRCA2-deficient cancer cells [49]. Upon breakdown of the micronuclear envelope [50], double-strand DNA (dsDNA) accumulates in the cytoplasm and is sensed by signalling pathways, such as by cyclic GMP-AMP (cGAMP) synthase (cGAS) [51]. cGAS activation produces cGAMP, which then activates stimulator of IFN genes (STING) and leads to the activation of TANK-binding kinase 1 (TBK1), IκB kinase (IKK) and NF-κB inducing kinase (NIK). Altogether, activation of these kinases results in the activation and nuclear translocation of IFN regulatory factor 3 (IRF3) and NF-κB, resulting in expression of type I IFN, interferon-stimulated genes (ISGs) and inflammatory cytokines [52]—further linking DDR with the immune system [6,53]. Mounting evidence suggests the chronic activation of cGAS/STING can paradoxically induce an immune suppressive TME that promotes tumour progression [52,54,55]. Accordingly, cGAS/STING activation pathway may exert either an antitumour or pro-tumour effect, depending on several factors, such as the stage of tumour progression and the tissue-specific context.

Through activation of the cGAS/STING pathway, PARPi appear to induce a CD8 T cell–dependent antitumour response. For instance, olaparib treatment induced a strong antitumour immune response in different pre-clinical tumour mouse models, including TNBC [56], SCLC [57] and BRCA1-deficient ovarian cancer [20], in a cGAS/STING-dependent manner. In the TNBC model, this effect is more pronounced in HR-deficient than in HR-proficient TNBC [56]. In addition, olaparib treatment leads to cGAS/STING-associated inflammatory response in BRCA2-deficient cells, which is further increased by ATRi [49,58]. Olaparib and rucaparib treatment modulate the immune response through the cGAS/STING pathway in ERCC1-defective, non-small cell lung cancer (NSCLC) and BRCA1-defective TNBC cells [44]. On the other hand, in an NSCLC mouse model, the PARPi niraparib plus radiation activated STING, resulting in antitumour immunity [59]. In addition, high concentrations of olaparib or talazoparib also activate STING in BRCA-proficient cells [21]. Recently, it has also been suggested that PARPi-mediated modulation of the immune response is dependent on the PARP trapping activity of PARPi [37].

Beyond PARPi, the impact of other DDR-targeted agents on the immune response to cancer mediated by the cGAS/STING pathway is beginning to be elucidated. For example, treatment with the CHK1i prexasertib increased the level of tumour-infiltrating T lymphocytes and synergises with anti-PD-L1 immunotherapy in a mouse model of SCLC [57]. Treatment with different ATRi results in innate immune and T cell activation mediated by cGAS-STING pathway in various mouse models of cancer, including those for prostate cancer [60], hepatocellular carcinoma [34] and ovarian cancer [61]. The combination of a WEE1i (adavosertib) and an ATRi (ceralasertib) also promotes accumulation of cytosolic dsDNA, which subsequently activates the STING pathway and induces the production of type I IFN and the recruitment and activation of CD8 T cells—thereby inducing antitumour immunity [62]. Similar induction of antitumour immunity through STING activation has been reported after treatment with ATMi [63,64]. In contrast, one study revealed that despite increased levels of cytoplasmic DNA, treatment with a WEE1i (adavosertib) and CHK1i (prexasertib, MK-8776 and PF-477736) fails to activate a type I IFN response [65].

Altogether, these pre-clinical studies suggest that DDR-targeted agents activate the immune system through STING activation, providing a rationale for combining these agents with immune response modulators such as immune checkpoint inhibitors (ICIs). However, it has recently been shown in a ovarian cancer model that tumour-intrinsic STING promotes resistance to dual ICI therapy via vascular endothelial growth factor A (VEGF-A) [55]. This highlights both the complexity of the interactions between tumour cells and the immune system as well as the importance of properly exploring these interactions. Given that STING activation can have both pro- and antitumour effects, further studies are needed to determine the synergy of these combinations in different situations.

### 3.2. Regulation of the Expression of Immune Checkpoint Interacting Molecules on Tumour Cells by DDR-Targeted Agents

A key way in which tumour cells can escape an immune response is by expressing immune checkpoint interacting molecules on their surface, including PD-L1 and PD-L2 [66]. Engagement of programmed death protein-1 (PD1), expressed on activated T lymphocytes [67], with their ligands PD-L1 or PD-L2, inhibits T cell activation [68,69] and results in failure of T cells to respond to tumours [70]. Accordingly, the blocking antibodies anti-PD-1, anti-PD-Ll and anti-PD-L2, which are defined as immune checkpoint inhibitors (ICI), have been developed to stop this engagement and allow proper T cell responses to tumours [71]. In addition to monoclonal antibodies, other strategies to block these interactions are being developed, including using soluble PD-1 receptors capable of binding and neutralizing both PD-L2 and PD-L1 [72] and small-molecule inhibitors targeting PD-1/PD-L1 signalling pathway [73].

PARPi upregulates expression of PD-L1 in different cancer cells, including breast [26,74], SCLC [57], NSCLC [16] and biliary tract cancer [75], and accordingly improve cancer-associated immunosuppression [74]. Different mechanisms have been proposed to explain the upregulation of PD-L1 by PARPi, such as: inhibition of glycogen synthase kinase 3β (GSK3β) [74], resulting in PD-L1 stabilization [76]; IFN-γ-induced PD-L1 expression [44]; and suppression of nucleophosmin (NPM1) interactions with PARP1, thereby enhancing the association of NPM1 at the PD-L1 promoter [77]. PD-L1 is also upregulated by other DDR-targeted agents besides PARPi. For instance, upregulation of PD-L1 was observed after the combination of a WEE1i (adavosertib) and an ATRi (ceralasertib) [62]. Although PD-L2 may also play a similar role as PD-L1, the effects of DDR-targeted agents on PD-L2 expression have not been explored so far.

The tumour evasion mechanism mediated by PD-L1 upregulation in response to DDR-targeted agents can be reverted by combining these agents with the aforementioned antibodies that block the interactions of PD-L1 with PD1 [22,56,60,74] or with agents that alter PD-L1 expression [78]. Numerous clinical trials have been initiated that combine DDR-targeted agents with ICIs [6,16,79]. The results of some of these phase II clinical trials (Table 1) are already beginning to emerge, with varied outcomes. For instance, combining durvalumab (an anti-PD-L1 antibody) with olaparib in patients with relapsed SCLC did not meet the pre-set bar for efficacy [14]. Similarly, olaparib plus durvalumab in recurrent ovarian cancer shows only a modest clinical response [80]. In contrast, in metastatic castration-resistant prostate cancer, the durvalumab/olaparib combination demonstrates clinical efficacy, particularly in patients with DDR abnormalities [81]. In germline BRCA1/2-mutated metastatic breast cancer, the durvalumab/olaparib combination shows promising antitumour activity and safety [82]. In addition, the triple combination of durvalumab, olaparib and paclitaxel shows superior efficacy compared to standard neoadjuvant chemotherapy in human epidermal growth factor receptor 2 (HER2)-negative breast cancer [83]. Similar results have been found for other combinations. For example, treatment of patients with advanced or metastatic TNBC with the PARPi niraparib plus the anti-PD1 antibody pembrolizumab has provided encouraging antitumour activity, with higher response rates in patients with tumour-promoting BRCA mutations [84]. In metastatic NSCLC, niraparib plus pembrolizumab also shows clinical activity [85]. In addition, the niraparib/pembrolizumab combination in patients with recurrent platinum-resistant ovarian carcinoma also shows promising antitumour activity [86].

Despite the aforementioned encouraging preliminary results from some clinical trials, we need to cautiously await the results of other ongoing clinical trials and evaluated whether addition of ICIs improves long-term clinical outcomes as compared with DDR-targeted agents monotherapy, to better evaluate their effectiveness [6,79]. Likewise, it is very important to advance in the identification of biomarkers that allow us to predict the clinical response to the combination of DDR-targeted agents and ICI [87].

### 3.3. Modulation of Other Ligands on Tumour Cells by DDR-Targeted Agents That Affect Their Interactions with Immune Cells

The impact of DDR-targeted agents in the modulation of other surface molecules on tumour cells that will play a role in their crosstalk with the immune system has been poorly explored. Two good examples of these kinds of molecules are death receptors and NKG2D ligands.

As mentioned above, NK cells can destroy tumour cells by inducing death receptor-mediated apoptosis upon engagement of TRAIL and FASL with their receptors, TRAIL receptor (TRAILR) and FAS, respectively, at tumour target cells [42]. Of note, PARPi treatment induces the expression of these death receptors on the surface of tumour cells, thereby sensitising these cells to death receptor-mediated apoptosis [88,89] (Figure 3).

NKG2D is an activating receptor expressed on the surface of different immune cells, including NK cells, which recognises ligands (NKG2DL) that are upregulated on tumour cells, resulting in NK cell-mediated recognition and cytolysis of target cells [90,91]. DNA damage induction in tumours cells upregulates NKG2DL for the co-stimulatory receptors NKG2D in a STING-dependent manner, thereby stimulating the cytotoxicity of NK cells [92]. Of note, the PARPi olaparib upregulates expression of NKG2DL on the surface of cells of a human acute myeloid leukaemia cell line [93]. However, doxorubicin-induced NKG2DL expression on multiple myeloma cells is abolished after treatment with ATMi and ATRi [94].

Further studies are required to define the different roles of DDR-targeted agents in modulating the expression of these receptors and their impact on the immune response to tumours. Interestingly, inhibition of WEE1 by adavosertib sensitises head and neck cancer cells to NK lysis by overcoming the resistance to granzyme B-induced cell death [95].

## 4. Concluding Remarks and Future Prospects

The approval of PARPi for clinical use in different tumour types has highlighted the usefulness of targeting DDR components for cancer treatment. Accordingly, numerous clinical trials are underway with agents targeting key components of DDR. Although the therapeutic rationale was initially based on their effect on tumour cells per se, recent developments have shown that these compounds can also modulate the interactions between tumour cells and their environment, and especially with components of the immune system. The effects of DDR-targeted agents on the immune system can be direct, acting on immune cells, or indirect, for instance by modifying components of tumour cells that are critical in their communication with components of the immune response. Modulation of the immune response by DDR-targeted agents can either promote the immune response to the tumour, or conversely block this response and promote tumour progression—as reviewed here, there is experimental data supporting both situations. This apparent dichotomy underscores the complexity of the interactions between tumour cells and the immune system, which can vary depending on numerous factors. Currently, there is considerable enthusiasm about the prospect of DDR-targeted agents in activating the immune response to tumours by targeting STING pathway activation. It is important to be cautious in this regard. Although the majority of pre-clinical studies using different DDR-targeted agents that have been published seem to support this hypothesis, how cGAS/STING activation affects tumour development is controversial, as it has been shown to either limit or favour tumourigenesis. Similarly, the combination of DDR-targeted agents with ICI is also attracting much attention, as indicated by the large number of clinical trials underway. Clearly, it is necessary to await the results of these long-term trials before drawing conclusions about the clinical potential of these combinations. In this regard, it may be very important to identify biomarkers that can predict clinical situations in which DDR-targeted agents and ICI combinations may be useful. Future work will need address the immunomodulatory roles of DDR-targeting agents, to help design and optimise novel therapeutics strategies in cancer.

## Figures and Tables

**Table 1:** Phase II clinical trials combining agents targeting DNA damage response with immune checkpoint inhibitors (ClinicalTrials.gov; accessed on 1 October 2021).

Study Identifier	Study Design	Condition	Target	Drug	Primary Outcome
NCT03955471	Single Arm	Ovarian Neoplasms	PARP	Olaparib + TSR-042 (anti-PD-1 mAb)	ORR (5 years)
NCT03951415	Single Arm	Endometrial Neoplasms, Uterine Neoplasms	PARP	Olaparib + Durvalumab (anti-PD-L1 mAb)	PFS (12 weeks)
NCT03851614	Two Arms	Mismatch Repair Proficient Colorectal Cancer, Pancreatic Adenocarcinoma, Leiomyosarcoma	PARP	Olaparib + Durvalumab (anti-PD-L1 mAb)	Changes in genomic and immune biomarkers (3 years)
NCT03780608	Single Arm with 2 Cohorts	Gastric Adenocarcinoma, Malignant Melanoma	ATR	Ceralasertib [AZD6738] + Durvalumab (anti-PD-L1 mAb)	ORR (2 years)
NCT03574779	Single Arm	Ovarian Neoplasms	PARP	Niraparib + TSR-042 (anti-PD-1 mAb) + Bevacizumab (anti-VEGF mAb)	ORR (6 years)
NCT03565991	Single Arm with 2 Cohorts	Advanced Solid Tumours with Defects in BRCA1/BRCA2, Advanced Solid Tumours with Defects in ATM	PARP	Talazoparib + Avelumab (anti-PD-L1 mAb)	OR (2 years)
NCT03330405	Two Arms with 10 Cohorts	Locally Advanced or Metastatic Solid Tumors	PARP	Talazoparib + Avelumab (anti-PD-L1 mAb)	ORR (2 years)
NCT03167619	Two Arms	Triple Negative Breast Cancer	PARP	Olaparib + Durvalumab (anti-PD-L1 mAb)	PFS (12 months)
NCT02953457	Single Arm	Recurrent ovarian, fallopian tube, or primary peritoneal cancer with BRCA1 or BRCA2 genetic mutation	PARP	Olaparib + Durvalumab (anti-PD-L1 mAb) + Tremelimumab (anti-CTLA-4 mAb)	PFS (3 and 6 months)
NCT02657889	Single Arm	Triple Negative Breast Cancer, Ovarian Cancer, Fallopian Tube Cancer, Peritoneal Cancer	PARP	Niraparib + Pembrolizumab (anti-PD-1 mAb)	ORR (40 weeks)
NCT02571725	Single Arm	Ovarian Cancer, Fallopian Tube Cancer, Peritoneal Neoplasms	PARP	Olaparib + Tremelimumab (anti-CTLA-4 mAb)	ORR (2 years)

mAb: monoclonal antibody; ORR: overall response rate; PFS: progression-free survival; OR: objective response.

## Data Availability

Not applicable.
